# Cavotricuspid isthmus ablation with a focal dual-energy catheter: First commercial experience

**DOI:** 10.1016/j.hroo.2025.11.017

**Published:** 2025-11-29

**Authors:** Mohamad Mdaihly, Arwa Younis, Andrew Lacombe, Shady Nakhla, Ayman Hussein, Bryan Baranowski, Pasquale Santangeli, Jakub Sroubek, Mandeep Bhargava, Walid Saliba, Mohamed Kanj, Oussama Wazni, David Martin, Arshneel Kochar, Koji Higuchi, Roy Chung, Tyler Taigen

**Affiliations:** Cardiac Electrophysiology and Pacing Section, Department of Cardiovascular Medicine, Cleveland Clinic, Cleveland, Ohio

**Keywords:** Cavotricuspid isthmus, CTI, Pulsed-field ablation, PFA, Radiofrequency, RF, Atrial flutter, AFL, Atrial fibrillation, AF

**Key Findings**

▪ A focal dual-energy mapping and ablation catheter (Sphere-9) enables efficient single-catheter workflow for combined atrial fibrillation and cavotricuspid isthmus (CTI) ablation, eliminating the need for catheter exchanges and supporting streamlined procedural management.
▪ CTI ablation performed with radiofrequency alone or in combination with pulsed-field ablation demonstrated acute efficacy, achieving 100% bidirectional block with high first-pass success and no procedural complications, including the absence of coronary spasm.
▪ Short-term outcomes were favorable, with minimal arrhythmia recurrence, supporting the feasibility and safety of this dual-energy strategy while underscoring the need for larger studies to confirm long-term durability.

Typical cavotricuspid isthmus (CTI) atrial flutter (AFL) frequently coexists with atrial fibrillation (AF). 20%–25% of patients with AF will also have documented typical AFL, and many treated for isolated AFL later develop AF.^1^ Thus, in patients with atrial arrhythmias, both pulmonary vein isolation (PVI) and CTI block may be required to achieve long-term freedom. Pulsed-field ablation (PFA) has become the preferred ablation modality for PVI, but its role in CTI remains limited.^2^^,^^3^ Currently, operators using PFA systems often switch to radiofrequency (RF) catheters for linear lesions such as the CTI line, adding time, cost, and complexity. Moreover, PFA delivery along the CTI can trigger severe right coronary artery spasm requiring intravenous nitroglycerin for prevention.^4^ In this study, we report our first commercial experience using a focal dual-energy mapping and ablation catheter (Sphere-9) for CTI ablation in patients undergoing catheter ablation for AF.

We reviewed 20 AF ablation procedures, in which CTI ablation was performed with the Sphere-9 catheter. Mapping and ablation were conducted using the Affera Prism-1 system under intracardiac echocardiographic (ICE) and fluoroscopic guidance. PVI and posterior wall ablation were performed using PFA (4-second applications), whereas CTI ablation was achieved with focal RF (75°C, 5 seconds) delivered in a linear overlapping fashion from the tricuspid annulus to the inferior vena cava. PFA was selectively applied in the posterior isthmus at the operator’s discretion, but avoided near the mid-anterior CTI to minimize right coronary artery spasm. When a mitral isthmus line was required for atypical AFL, RF and PFA were combined per operator’s judgment. Bidirectional CTI block was confirmed with differential pacing using the Sphere-9 catheter positioned laterally and a proxim"al coronary sinus catheter medially. This study was approved by the Cleveland Clinic’s Institutional Review Board. Because this was a retrospective study using electronic patient records only, informed consent was not required by the institutional review board.

20 patients (mean age 69.4 ± 8.4 years; 13 (65%) male; mean CHA_2_DS_2_-VASc score 3.4 ± 1.8) underwent combined AF and CTI ablation. 16 (80%) had persistent AF, and 18 (90%) had a history of typical AFL. 13 (65%) had undergone AF ablation, and 1 (5%) had undergone remote CTI ablation. PVI was performed in all patients, with 9 (45%) also requiring a mitral isthmus line for atypical AFL substrate. 3 (15%) patients had cardiac electronic devices implanted, with no postprocedural changes observed in device performance.

**Figure 1:**
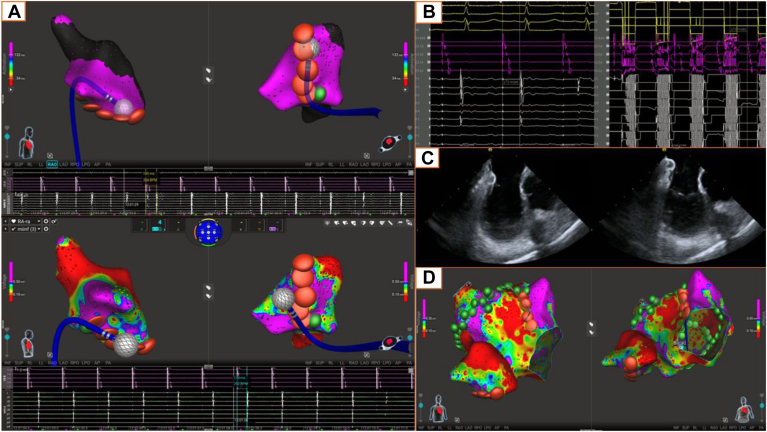
**A:** Top: Sphere-9 catheter positioned directly on the CTI ablation line with observed local split potentials (185 ms) postablation. Bottom: Final CTI ablation map with RF lesions (*red*) and PFA lesions (*green*) superimposed on the baseline right atrial voltage map. PFA was performed only at the posterior CTI near the inferior cavoatrial junction, far from the right coronary artery. **B:** Differential atrial pacing using a proximal coronary sinus catheter and Sphere-9 catheter positioned just lateral to the CTI ablation line. The postablation isthmus conduction time is 170 ms in both directions, consistent with bidirectional CTI block (the preablation isthmus conduction time was 66 ms). **C:** Intracardiac echocardiography images acquired during CTI ablation, with the Sphere-9 catheter located on the CTI. **D:** Baseline right and left atrial voltage maps with the corresponding RFA and PFA lesions. In total, 85 PFA lesions and 10 RF lesions were applied to complete redo pulmonary vein isolation and posterior wall isolation (PFA), create an anterior mitral line with RFA + PFA, a CTI line (RFA + PFA), and superior vena cava isolation (PFA; not shown). CTI = cavotricuspid isthmus; PFA = pulsed-field ablation; RFA = radiofrequency ablation.

Bidirectional CTI block was achieved in all 20 patients, confirmed by split electrograms across the line and a mean postablation transisthmus conduction time of 180.3 ± 23 ms. The time from the first to the final lesion to establish block was 8.8 ± 6.4 minutes. RF alone completed the line in 13 (65%) patients (13.5 ± 6.4 applications), whereas 7 (35%) received supplemental PFA (2.0 ± 1.2 applications). Of these, 2 (28.6) patients received a single PFA lesion along the posterior CTI near the inferior cavoatrial junction (Figure 1), and in the remaining 5 (71.4%), PFA was applied away from the right coronary artery under ICE guidance. In 18 (90%) patients, bidirectional block was achieved with the first-pass linear lesion along the CTI; the other 2 (10%) required additional RF lesions approximately mid-CTI to achieve block. No complications were observed, including no coronary artery spasm, ischemic electrocardiographic changes, change in atrioventricular conduction times, hypotension, or need for vasodilators. No patient was treated with nitroglycerin or required vasopressor support during CTI ablation.

At a mean follow-up of 138.5 days, 2 (10%) patients experienced arrhythmia recurrence, neither of whom had typical right AFL. One developed AF during the blanking period, which persisted and required cardioversion. Another had atypical AFL recurrence, which required cardioversion on 2 occasions and the initiation of dofetilide.

This experience highlights the feasibility of using a single focal dual-energy catheter for combined AF and AFL ablation. By applying PFA in the left atrium and RF at the CTI, the workflow was streamlined without catheter or platform exchanges. Acute efficacy was excellent, with all CTI lines achieving bidirectional block. The catheter’s smaller and maneuverable focal design is better suited for CTI anatomy, while its conformable spherical lattice and slightly larger effective lesion footprint than that of a conventional RF tip, together with excellent stability and ICE visualization, facilitate the creation of a continuous linear CTI lesion.

Furthermore, CTI ablation was performed primarily with RF, with no complications observed and no patients requiring nitroglycerin or additional vasopressors. This contrasts with prior reports where focal PFA at the CTI, delivered without nitroglycerin, induced coronary spasm in up to 80% of patients.^5^ Accordingly, PFA is best reserved for established targets such as PVI whereas RF remains preferred for typical flutter, given limited data and PFA risks in this setting.

Importantly, this dual-energy strategy provides reliable confirmation of durable CTI block, avoiding the uncertainty sometimes seen with PFA-only approaches, such as coronary spasm, atrioventricular conduction changes, and demonstration of acute block, or even less dependable long-term durability. Our experience demonstrates favorable short-term outcomes with Sphere-9 for combined AF and AFL ablation. However, larger studies with longer follow-up are warranted to confirm lesion durability and long-term outcomes.
